# Isolation and characterization of the compounds responsible for the antimutagenic activity of *Combretum microphyllum* (Combretaceae) leaf extracts

**DOI:** 10.1186/s12906-017-1935-5

**Published:** 2017-09-06

**Authors:** Tshepiso Jan Makhafola, Esameldin Elzein Elgorashi, Lyndy Joy McGaw, Maurice Ducret Awouafack, Luc Verschaeve, Jacobus Nicolaas Eloff

**Affiliations:** 1 Phytomedicine Programme, Department of Paraclinical Sciences, Faculty of Veterinary Science, University of Pretoria, Private Bag X04, Onderstepoort, 0110 South Africa; 2 Toxicology and Ethnoveterinary Medicine, Food, Feed and Veterinary Public Health, ARC-Onderstepoort Veterinary Institute, Private Bag X05, Onderstepoort, 0110 South Africa; 3 Department of Paraclinical Sciences, Faculty of Veterinary Science, Private Bag X04, Onderstepoort, 0110 South Africa; 4 Scientific Institute of Public Health, Rue Juliette Wytsmanstreet 14, 1050 Brussels, Belgium; 5 Department of Biomedical Sciences, University of Antwerp, Universiteitsplein 1, B-2610 Wilrijk, Belgium

**Keywords:** *Combretum microphyllum*, n-Tetracosanol, Eicosanoic acid, Arjunolic acid, Antimutagenicity, Cytotoxicity, Antioxidant activity

## Abstract

**Background:**

Mutations play a major role in the pathogenesis and development of several chronic degenerative diseases including cancer. It follows, therefore that antimutagenic compound may inhibit the pathological process resulting from exposure to mutagens. Investigation of the antimutagenic potential of traditional medicinal plants and compounds isolated from plant extracts provides one of the tools that can be used to identify compounds with potential cancer chemopreventive properties. The aim of this study was to isolate and characterise the compounds responsible for the antimutagenic activity of *Combretum microphyllum*.

**Methods:**

The methanol leaf extract of *C. microphyllum* was evaluated for antimutagenicity in the Ames/microsome assay using *Salmonella typhimurium* TA98. TA100 and TA102. Solvent-solvent fractionation was used to partition the extracts and by using bioassay-guided fractionation, three compounds were isolated. The antimutagenic activity of the three compounds were determined in the Ames test using *Salmonella typhimurium* TA98, TA100 and TA102. The antioxidant activity of the three compounds were determined by the quantitative 2,2-diphenyl-1-picrylhydrazyl (DPPH)-free radical scavenging method. The cytotoxicity was determined in the MTT assay using human hepatocytes.

**Results:**

A bioassay-guided fractionation of the crude extracts for antimutagenic activity led to the isolation of three compounds; n-tetracosanol, eicosanoic acid and arjunolic acid. Arjunolic acid was the most active in all three tested strains with a antimutagenicity of 42 ± 9.6%, 36 ± 1.5% and 44 ± 0.18% in *S. typhimurium* TA98, TA100 and TA102 respectively at the highest concentration (500 μg/ml) tested, followed by eicosanoic acid and n-tetracosanol. The antioxidant activity of the compounds were determined using the quantitative 2,2 diphenyl-1-picryhydrazyl (DPPH)-free radical scavenging method. Only arjunolic acid had pronounced antioxidant activity (measured as DPPH-free scavenging activity) with an EC_50_ value of 0.51 μg/ml. The cytotoxicity of the isolated compounds were determined in the MTT assay using human hepatocytes. The compounds had low cytotoxicity at the highest concentration tested with LC_50_ values >200 μg/ml for n-tetracosanol and eicosanoic acid and 106.39 μg/ml for arjunolic acid.

**Conclusions:**

Based on findings from this study, compounds in leaf extracts of *C. microphyllum* protected against 4-NQO and MMC induced mutations as evident in the Ames test. The antimutagenic activity of arjunolic acid may, at least in part, be attributed to its antioxidant activity resulting in the detoxification of reactive oxygen species produced during mutagenesis.

**Electronic supplementary material:**

The online version of this article (10.1186/s12906-017-1935-5) contains supplementary material, which is available to authorized users.

## Background

Mutations are implicated in the etiopathology of cancer, neurodegenerative diseases and several other chronic degenerative diseases. They are caused by permanent transmissible changes in the DNA structure and may involve individual genes, blocks of genes or whole chromosomes [1]. Since mutagens are involved in the initiation and promotion of several human diseases, research focusing on the identification of novel bioactive phytocompounds that reduce mutagenicity and counteract mutagenesis has gained credence in recent years [2]. The possibility of moderating the response of cells to a particular mutagen by antimutagenic compounds opens new horizons in the prevention of chronic degenerative diseases. Antimutagens provide multiple points of intervention for the pharmacological prevention of mutation related diseases. They are involved in the prevention of mutations and cancer development by lowering the frequency and/or rate of mutations, or blocking initiation of carcinogenesis; a chemopreventive role [2, 3]. Induction of mutagenesis occurs mainly through damage of DNA by free radicals and other reactive oxygen species (ROS) [4]. Numerous mutagens act through generation of ROS. Antioxidants which are inhibitors of oxidation are therefore an important part of a strategy to minimize mutation related diseases by prevention of oxidation induced DNA damage [5].

Plants synthesize structurally varied biologically active secondary metabolites with therapeutic potential as well as antimutagenic and anticarcinogenic properties [6]. On this basis, the search for antimutagens from plants presents possibilities for the discovery of new antimutagenic and anticarcinogenic phytocompounds. Furthermore, there is generally a growing interest in research relating to chemoprevention of cancer and other mutation related diseases [7]. Species of the Combretaceae family are widely spread and used for medicinal purposes in traditional medicine in different continents in the world. A review paper on the biological activity and chemistry of southern African Combretaceae identified gaps and areas for future research [8]. Different extractants extract different compounds with differing biological activity from *Combretum microphyllum* [9]. After investigating the correlation between antioxidant activity and antimutagenic activity of extracts of 120 plant species we found that *C. microphyllum* leaf extracts had high antimutagenic activity based on the Ames test, micronucleus/cytome assay and comet assay [10]. The aim of this study was to isolate and characterise the antimutagenic compounds present in *C. microphyllum* leaf extracts using bioassay-guided fractionation.

## Methods

### Plant material collection

Leaves of *Combretum microphyllum* Klotzsch were collected from the Lowveld National Botanical Gardens in Nelspruit, South Africa. A herbarium and voucher specimen number (Lowveld NBG 259/1995) was deposited at the Lowveld National Botanical Garden Herbarium in Nelspruit, South Africa. The leaves were dried in the dark at room temperature, ground into a fine powder and stored in glass bottles in the dark until used.

### Extraction and bioassay-guided fractionation of compounds

The powdered leaves (580 g) were extracted three times with 5 l of methanol overnight. The extract was filtered through Whatman No.1 filter paper and concentrated to dryness using a rotary evaporator. The crude extract yield was 120.98 g. the crude extract was subjected to solvent-solvent fractionation using 1.2 l of *n-*hexane, ethyl acetate, *n*-butanol and water respectively. The resulting fractions were concentrated to dryness with a rotary evaporator and decanted into preweighed bottles and placed overnight under a stream of cold air to for solvent evaporation. The fractions were kept at 4 °C. The antimutagenic activity of the fractions was determined using the Ames test as an indicator of antimutagenicity. The most active ethyl acetate fraction (27.8 g) was subjected to open column chromatography (CC) on silica gel 60 (Merk) and eluted with an increasing polarity system of hexane and ethyl acetate [90:10 to 0:100] at 10% increments. Fractions of volume 300 ml each were collected and combined based similarity of thin layer chromatograms.

### Determining mutagenicity and antimutagenicity

The potential mutagenic and antimutagenic effects of the isolated compounds were determined using the *Salmonella*/microsome (Ames test) assay [11], performed with *Salmonella typhimurium* TA98, TA100 and TA102. 4-Nitroquinoline 1-oxide and mitomycin C were used as positive controls. Briefly, 100 μl of bacterial stock were incubated in 20 ml of Oxoid Nutrient broth for 16 h at 37 °C on a rotary shaker. Of this overnight culture, 0.1 ml was added to 2.0 ml of top agar (containing histidine-biotin) together with 0.1 ml test solution and 0.5 ml phosphate buffer. To determine mutagenicity, the test solution contained 50 μl test sample and 50 μl solvent control. To determine antimutagenicity, the test solution contained 50 μl test sample and 50 μl positive control). The top agar mixture was poured over the surface of a minimal agar plate and incubated for 48 h at 37 °C. After incubation the numbers of revertant colonies (mutants) in each plate were counted.

Antimutagenicity was expressed as percentage inhibition of mutagenicity calculated using the formula below:

$$ \%\mathrm{inhibition}=\left[1-\left(\frac{T}{M}\right)\right]\times 100 $$

Where T is the number of revertants per plate in the presence of mutagen and the test solution and M is the number of revertants per plate in the positive control. All cultures were prepared in triplicate (except for the solvent control where five replicates were used). Absence of toxicity was confirmed when a background layer of bacterial growth, which should normally be present was observed. The positive control for TA98 and TA100, 4-nitroquinoline 1-oxide (4-NQO), was used at concentrations of 2 μg/ml and 1 μg/ml respectively, and for TA102, the positive control MMC was used at 1 μg/ml.

### Quantitative antioxidant activity

The DPPH free radical scavenging spectrophotometric method described by Mensor et al. [12] and modified by Aderogba et al. [13] was used to evaluate the quantitative antioxidant activity. Reactions were carried out in 96-well microtitre plates and each of the isolated compounds was tested at varying concentrations ranging from 100 to 0.048 μg/ml. Blank solutions were prepared with methanol only while the negative control was DPPH solution (20 μl plus 50 μl methanol). Test sample solution contained compounds serially diluted in methanol. Methanol served as a blank for the microplate reader and the decrease in absorbance was measured at 515 nm. Percentage antioxidant activity (AA%) values were calculated from the absorbance values using the formula:

$$ \mathrm{AA}\%=100-\left\{\left[\left(\mathrm{Abs}\ \mathrm{sample}\hbox{--} \mathrm{Abs}\ \mathrm{blank}\right)\times 100\right]/\mathrm{Abs}\ \mathrm{control}\right\} $$

(Abs sample is the absorbance of the sample, Abs blank is the absorbance of the blank and Abs control is the absorbance of the control). L-ascorbic acid (vitamin C) was used as a positive control (antioxidant agent). The EC_50_ value, defined as the concentration of the sample leading to 50% reduction of the initial DPPH concentration, was calculated from the separate linear regression of plots of the mean percentage of the antioxidant activity against concentration of the test extracts obtained from the three replicate assays. The results are expressed as EC_50_ values obtained from the regression plots.

### Tetrazolium-based cytotoxicity test (MTT assay)

Cytotoxic effects of the isolated compounds were determined using the tetrazolium-based colorimetric (MTT) assay against human hepatocellular carcinoma (C3A) cells using the method described by Mosmann [14]. Briefly, the cells were maintained in minimal essential medium supplemented with 10% foetal calf serum and sodium pyruvate. Cell suspensions were prepared from confluent monolayer cultures and plated at a density of 5 × 10^4^ cells/ml and a total of 200 μl of the cell suspension was plated in a 96-well culture plate. After incubation at 37 °C in a 5% CO_2_ incubator, the cells were treated with different concentrations of the isolated compounds ranging from 10 to 200 μg/ml and incubated for 2 days. Doxorubicin chloride was used as the positive control. The wells were washed with PBS and fresh medium (200 μl) was then added to the wells. MTT (Sigma) dissolved in PBS (30 μl) was added to each well and incubated for 4 h at 37 °C. The medium was removed and MTT formazan crystals were dissolved in 50 μl DMSO. The amount of MTT reduction was measured immediately by detecting the absorbance using a microplate reader (BioTek Synergy, Analytical and Diagnostic Products, South Africa) at a wavelength of 570 nm. The percentage of cell viability was calculated using the formula below:

$$ \%\mathrm{cell}\  \mathrm{viability}\kern0.5em =\kern0.5em \left[\frac{\mathrm{Mean}\  \mathrm{Absorbance}\  \mathrm{of}\  \mathrm{sample}}{\mathrm{Mean}\  \mathrm{Absorbance}\  \mathrm{of}\  \mathrm{control}}\right]\times 100 $$

The LC_50_ values were calculated as the concentration of the test sample that resulted in a 50% reduction of absorbance compared to untreated cells. The intensity of the MTT formazan produced by living metabolically active cells is directly proportional to the number of live cells present (Mosmann, [14]).

## Results and discussion

**Fig. 1:**
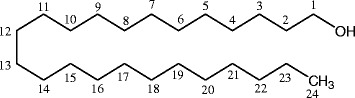
Chemical structure of n-tetracosanol

**Fig. 2:**
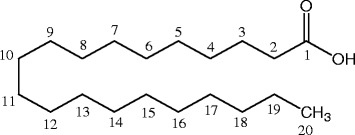
Chemical structure of eicosanoic acid

**Fig. 3:**
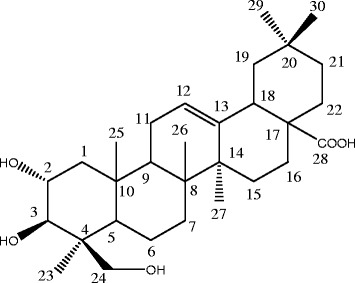
Chemical structure of arjunolic acid

Bioassay-guided liquid-liquid fractionation of the crude methanol extract of the dried leaves of *C. microphyllum* using column chromatography and determining antimutagenic activity yielded three compounds. Compound 1 (12 mg) was obtained as a powder, Compound 2 (11.3 mg) was obtained as a white powder and compound 3 (15 mg) was obtained by repeated column chromatography purification until single spots were obtained in TLC. (NMR spectra provided in the Additional file 1). The compounds were identified as n-tetracosanol (C1), eicosanoic acid (C2) and arjunolic acid (C3) using ^1^H and ^13^C NMR spectroscopic analysis. The structures were confirmed by comparison of the NMR data obtained with data in the literature: n-tetracosanol (Fig. 1) [15], eicosanoic acid (Fig. 2) [16, 17] and arjunolic acid (Fig. 3) [18, 19].

The mutagenic activities of the three compounds isolated from *C. microphyllum* are presented in Table 1. The three compounds had no mutagenic activity in the Ames test using *S. typhimurium* TA98, TA100 and TA102. The compounds did not induce a significant increase (*p*˃0.05) in the number of revertant colonies compared to the negative control (solvent blank). There was a significant difference (*p*˂0.001) in the number of revertant between the positive control when compared to the negative control and all three compounds; a clear indication on the sensitivity of the assay in detecting mutagens. The mutation frequency/index for all the three strains when exposed to differing concentrations of the isolated compounds was less than 2, meaning none of the extracts caused double the number of colonies compared to the negative control. A positive mutagenic response in the Ames test requires at least a doubling in the number of revertant colonies of the test sample compared to the negative control [20] For all three compounds tested in the current experiments, the density of background bacterial lawn was compared to that of the negative control (after 48 h) and found to have no visible differences, indicating a lack of toxicity to the bacteria at the concentration tested [11].

**Table 1:** Mean number of revertant colonies per plate (±SD) in *Salmonella typhimurium* TA98, TA100 and TA102 exposed to different concentrations of the compounds isolated from *C. microphyllum* to measure mutagenicity

Concentration μg/ml	500	50	5
*S. typhimurium* TA98			
n-Tetracosanol	30.00 ± 7.81	35.67 ± 9.71	23.67 ± 4.51
Eicosanoic acid	27.77 ± 1.53	29.33 ± 4.04	28.67 ± 5.51
Arjunolic acid	33.00 ± 2.65	28.33 ± 3.78	26.67 ± 1.15
Negative/solvent blank	28.60 ± 5.32	Positive 2 μg/ml 4-NQO	239.33 ± 33.20
*S. typhimurium* TA100			
n-Tetracosanol	125.00 ± 8.18	121.33 ± 2.52	127.00 ± 7.21
Eicosanoic acid	108.67 ± 5.03	102.67 ± 4.73	112.33 ± 2.89
Arjunolic acid	109.00 ± 8.72	104.33 ± 2.52	107.33 ± 1.15
Negative/solvent blank	107.00 ± 4.85	Positive 1 μg/ml 4-NQO	864.00 ± 9.77
*S. typhimurium* TA102			
n-Tetracosanol	294.33 ± 20.74	271.00 ± 4.58	286.67 ± 8.50
Eicosanoic acid	292.33 ± 5.51	278.33 ± 7.57	288.00 ± 10.82
Arjunolic acid	287.00 ± 15.39	280.67 ± 10.69	288.67 ± 28.68
Negative/solvent blank	282.40 ± 15.53	Positive 1 μg/ml MMC	1241.67 ± 7.77

**Fig. 4:**
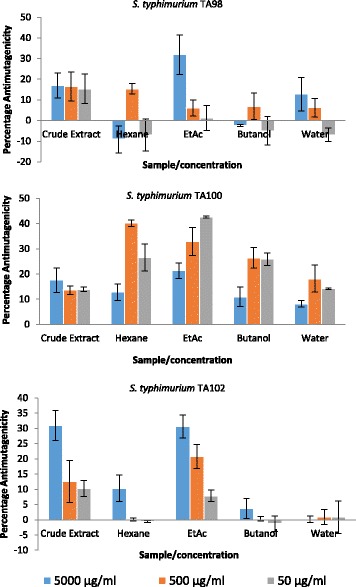
Antimutagenic activity of the crude extract of *C. microphyllum* and solvent-solvent fractions of the crude extract in the Ames test using *S. typhimurium* TA98, TA100 and TA102 (percentage inhibition of the mutagenic effects of 4-NQO and MMC). C1 = n-Tetracosanol, C2 = Eicosanoic acid and C3 = Arjunolic acid

The antimutagenic activity of the methanol crude extracts of *C. microphyllum* and solvent-solvent fractions of the crude extract are presented in Fig. 4 as percentage inhibition of the mutagenic effects of 4-NQO and MMC. Varying degrees of antimutagenicity were observed across all tester strains. The ethyl acetate fraction was the most active fraction in all tester strains. The activity significantly increased with an increase in concentration (*p*˂0.001 in the case of *S. typhimurium* TA102) and in some cases more active than the crude extract It was for this reason that it was selected for further fractionation and possible isolation of antimutagenic compounds.

**Fig. 5:**
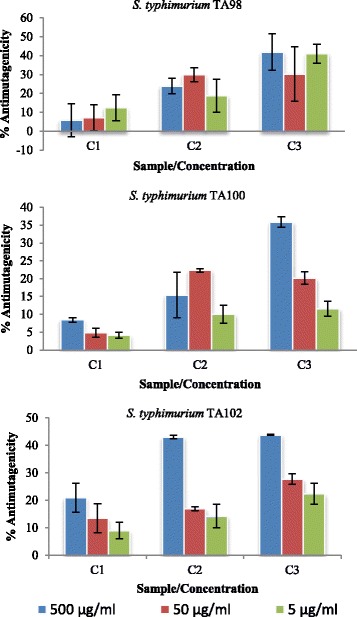
Antimutagenic activity of compounds isolated from *C. microphyllum* in the Ames test using *S. typhimurium* TA98, TA100 and TA102 (percentage inhibition of the mutagenic effects of 4-NQO and MMC). C1 = n-Tetracosanol, C2 = Eicosanoic acid and C3 = Arjunolic acid

The antimutagenic activity results of the three compounds isolated from the ethyl acetate fraction of *C. microphyllum* are presented in Fig. 5. All the compounds had antimutagenic activity in the Ames test. Even though the activity of the compounds was not significantly different in *S. typhimurium* TA98, there was a clear statistical difference in the activity of all three compounds in *S. typhimurium* TA100 and TA102 tester strains. The compounds clearly have multiple mechanisms of mutation inhibition as they inhibit mutagenicity of 4-NQO in *S. typhimurium* TA98, *S. typhimurium* TA100 and of MMC in *S. typhimurium* TA102. Moreover, these compounds may have varying mechanisms of antimutagenesis since they prevent frame-shift mutations detectable in TA98, base-pair substitutions detectable in TA100 and small in-frame deletions detectable in TA102. This is one of the many advantages of using the Ames test in antimutagenesis studies as it provides information not only of antimutagenesis but also on possible mode of action [21].

Arjunolic acid was the most active in all three tested strains with percentage antimutagenicity of up to 41.92 ± 9.59%, 35.84 ± 1.45% and 43.78 ± 0.18% in *S. typhimurium* TA98, TA100 and TA102 respectively at the highest concentration tested (500 μg/ml), followed by eicosanoic acid and n-tetracosanol. The compounds had better activity than the crude extract and the solvent-solvent fractions (Figs. 4 and 5). The compounds had at least a 10 times higher antimutagenic activity than the crude extract and the fractions. This is in agreement with reports that antimutagenicity of plants is caused by a small amount of a highly active compound or a large quantity of a weakly active agent or by the cumulative effect of many components [6].

Arjunolic acid effectively reduced the DPPH free radical with an EC_50_ value of 6.25 ± 0.29 μg/ml compared to the EC_50_ value of 0.51 ± 0.08 μg/ml of the positive control ascorbic acid (Table 2). This compound had significantly higher activity when compared to both n-tetracosanol and eicosanoic acid (*p* ˂0.001). The antioxidant activity of arjunolic acid observed in this study is in agreement with results reported by Manna et al. [22] where high levels of antioxidant activity were recorded at concentrations ranging from 100 to 600 μg/ml in a cell-free system. They recorded up to 80% DPPH free radical scavenging activity of arjunolic acid at the lowest concentration of 100 μg/ml used. The scavenging properties of this compound serve as a clear indication of its antioxidant potential. Based on this observation, the antimutagenic activity of arjunolic acid, at least in part, may be attributed to its antioxidant activity resulting in the detoxification of reactive oxygen species produced during mutagenesis. Arjunolic acid contains polyhydroxyl groups and thus can easily be oxidised during its interaction with reactive oxygen species (ROS). The DPPH radical scavenging activity of arjunolic acid can further be explained by the presence of its carboxylic hydrogen atom that can easily be abstracted by any free radical like DPPH [22].

**Table 2:** DPPH free radical scavenging (antioxidant) activity of three compounds isolated from *C. microphyllum*

Compounds	n-Tetracosanol	Eicosanoic acid	Arjunolic acid	Ascorbic acid
EC_50_ (μg/ml)	>100	>100	6.25 ± 0.29	0.51 ± 0.08

The cytotoxicity of n-tetracosanol, eicosanoic acid and arjunolic acid was assessed in the MTT assay using human liver cells. The results are presented in Table 3 as LC_50_ values in μg/ml. All three compounds had low cytotoxicities with LC_50_ values >200 μg/ml for n-tetracosanol and eicosanoic acid and 106.39 ± 5.11 μg/ml for arjunolic acid. The percentage cell viability for each compound at the highest concentration tested (200 μg/ml) was: 59.74 ± 7.23% and 50.09 ± 6.21% for n-tetracosanol and eicosanoic acid respectively. Ramesh and colleagues (2012) also found arjunolic acid to be toxic to Ehrlich ascites carcinoma (EAC) and Dalton’s lymphoma (DLA) cell lines. In their investigations, arjunolic acid inhibited cell growth by up to 70% at 100 μg/ml whilst in our present study arjunolic acid inhibited 66% of hepatocellular carcinoma C3A cell growth at 200 μg/ml. Based on these findings, it appears that the cytotoxic effects of arjunolic acid may be cell line specific.

**Table 3:** Cytotoxicity of three compounds isolated from *C. microphyllum* against human liver cells (C3A cell line)

Compounds	n-Tetracosanol	Eicosanoic acid	Arjunolic acid	Doxorubicin
LC_50_ (μg/ml)	>200	>200	106.39 ± 5.11	0.64 ± 0.032 μM

It is evident that indeed aliphatic alcohols have low cytotoxicity as found for n-tetracosanol in this study. Most aliphatic alcohols are not cytotoxic at concentrations of up to 300 mM [23]. These compounds are expected to have no adverse hepatotoxic effects. Most reports of toxic effects due to the use of herbal medicines and dietary supplements are associated with hepatotoxicity, although reports of ther toxic effects including kidney, nervous system, blood, cardiovascular and dermatologic effects, mutagenicity and carcinogenicity have also been published [24].

n-Tetracosanol, an aliphatic alcohol with 24 carbons, was the least active compound in all the tester strains. Nonetheless, the antimutagenic activity of this compound to some extent may be correlated to the activity of other aliphatic alcohols reported in literature. Aliphatic alcohols are known to have various biological activities. C18 to C26 aliphatic alcohols have antiproliferative activity on hyper-proliferative skin lesions. These compounds had selective antiproliferative activity against hypertrophic fibroblasts [25]. It is a central premise of medicinal chemistry that structurally similar molecules have similar biological activities [26]. There is a direct correlation between related chemical compounds and compositions and their therapeutic activities [23].

Arjunolic acid is a triterpenoid and a major constituent present in *Terminalia arjuna* [27, 28]. Arjunolic acid was isolated from the ethyl acetate fraction and methanol extracts of *T. arjuna* core wood [19]. There is no previous report on the antimutagenic activity of arjunolic acid. Hemalatha et al. [29] did however report that arjunolic acid has antimutagenic activity in a review article on the multifunctional therapeutic applications of arjunolic acid. No data was, however provided to support this conclusion, making our study the first to demonstrate antimutagenicity of arjunolic acid in the Ames test. Ever since the registration of a patent on hormonal, wound healing and bactericidal properties of arjunolic acid by Ratsimamanga and Boiteau [30] various biological activities of this compound have been studied [28]. Arjunolic acid has multi-functional medicinal applications including antioxidant, antiplatelet, anticoagulant, antinecrotic, anti-tumour, antinephrotoxic, antihepatotoxic, anti-inflammatory, anti-nociceptive, anticholinesterase, antidiabetic, anti-asthmatic, antimicrobial and anti-insecticidal activities [29, 31].

## Conclusion

The antimutagenic activity of *C. microphyllum* extracts, fractions and isolated compounds in protecting against 4-NQO and MMC induced mutations as evident in the Ames test was demonstrated for the first time in this study. The active antimutagenic constituents were n-tetracosanol, eicosanoic acid and arjunolic acid. The isolated compounds had varying antimutagenic activity, antioxidant activity and did not have substantial toxicity towards human liver cells (C3A cell line). Arjunolic acid was the only compound with good antioxidant activity and had better antimutagenic activity than eicosanoic acid and n-tetracosanol. The antimutagenic activity of arjunolic acid may, at least in part, may be attributed to its antioxidant activity resulting in the detoxification of reactive oxygen species produced during mutagenesis.

## Additional file

Additional file 1: Supplementary data is attached. (DOCX 1989 kb)

## Abbreviations

4-NQO: 4-nitroquinoline 1-oxide
CC: Column chromatography
DNA: Deoxyribonucleic acid
DPPH: 2, 2-diphenyl-1-picrylhydrazyl
EC_50_: Effective concentration
MMC: Mitomycin-C
MTT: [3-(4,5-dimethylthiazol-2-yl)-2,5-diphenyltetrazolium bromide]
ROS: Reactive oxygen species
TLC: Thin layer chromatography
