# Supplementary material for: Isolation and characterization of the compounds responsible for the antimutagenic activity of Combretum microphyllum (Combretaceae) leaf extracts
Source: BMC Complement Altern Med. 2017 Sep 6;17:446. doi: 10.1186/s12906-017-1935-5 (PMC5585923; doi:10.1186/s12906-017-1935-5)
Supplement: Additional file 1: — Supplementary data is attached. (DOCX 1989 kb) [file 12906_2017_1935_MOESM1_ESM.docx]

**Antimutagenicity, antioxidant activity and cytotoxicity of n-tetracosanol, eicosanoic acid and arjunolic acid Isolated from Combretum microphyllum (Combretaceae)**

**Tshepiso Jan Makhafola , Esameldin Elzein Elgorashi , Lyndy Joy McGaw ,** **Maurice Ducret Awouafack, Luc Verschaeve ^5^ and Jacobus Nicolaas Eloff^*^**

**Additional file 1**

Compound 1 (n-tetracosanol) was obtained as a white powder (12 mg). The NMR spectra (^1^H, ^13^C, ASAP-HMQC, COSY and HMBC) are presented in Figures 1-5. The ^1^H-NMR spectrum (Table 1) had four sets of proton signals at *δ* 3.64 (br.*t*, 4.0, 8.0 Hz, 2H), 1.56 (*m*, 2H), 1.25 (br.*s*, 42H) and 0.88 ppm (*t*, 8.0 Hz, 3H) corresponding to protons at position C-1, C-2, C-3 – C-23 and C-24, respectively. The ^13^C-NMR spectrum (Table 5.2, Figure 5.8) had characteristic signals for a fatty acid derivative at *δ* 63.1 (CH_2_), 32.8 (CH_2_), 31.9 (21CH_2_) and 14.1 (CH_3_) ppm corresponding to an oxygenated methylene (C-1), a methylene (C-2), a methylenic side chain (C-3 - C-23) and a methyl (C-24) groups, respectively. The analysis of the spectroscopic data (^1^H-, ^13^C-NMR, HMQC, COSY and HMBC) compared with those reported in the literature enabled its unambiguous identification as n-tetracosanol (Fig 1-5) [7].

Table S1 ^1^H- (400 MHz) and ^13^C- (100 MHz) NMR data of n-tetracosanol in CDCl_3_, (*δ* in ppm, *J* in Hz)

| n-Tetracosanol | | |
| --- | --- | --- |
| Position | **^13^C** | **^1^H** |
| 1 | 63.1, (CH_2_) | 3.64, br*.t* (4.0, 8.0), 2H |
| 2 | 32.8, (CH_2_) | 1.56, *m*, 2H |
| 3-23 | 31.9 – 22.7, (CH_2_) | 1.25, br*.s*, 42H |
| 24 | 14.1, (CH_3_) | 0.88, *t*, (8.0) 3H |

Compound 2 (eicosanoic acid) was obtained as a white powder (11.3 mg). The NMR spectra (^1^H, ^13^C, ASAP-HMQC, COSY and HMBC) are presented in Figure 6-10. The ^13^C-NMR spectrum (Table 2, Figure 7) had a characteristic signal at *δ* 178.1 ppm assignable to a carboxylic group of a fatty acid. The presence of an acid group was substantiated by the broad singlet observed on the ^1^H-NMR spectrum (Table 2) at *δ* 10.00 ppm corresponding to the proton of the hydroxyl group. Others signals were observed on the ^13^C-NMR spectrum at *δ* 33.7 (CH_2_), 31.9 (CH_2_), 29.7 - 22.7 (16 CH_2_) and 14.1ppm (CH_3_) corresponding to carbons at positions C-2, C-3, C-4 – C-19 and C-20, respectively. Similar signals as those from compound **1** described above were observed on the ^1^H-NMR spectrum (Table 2, Figure 6) at *δ* 2.35 (br.*t*, 4.0, 8.0 Hz, 2H), 1.63 (*m*, 2H), 1.25 (br.*s*, 32H) and 0.88 ppm (*t*, 8.0 Hz, 3H), and corresponding to protons at positions C-2, C-3, C-4 – C-19 and C-20, respectively. All these data were in agreement with those of eicosanoic acid (**2**), also called arachidic acid, previously isolated from *Milletia laurenti* [8, 9].

Table S2 ^1^H- (400 MHz) and ^13^C- (100 MHz) NMR data of eicosanoic acid in CDCl_3_, (*δ* in ppm, *J* in Hz)

| Eicosanoic acid | | |
| --- | --- | --- |
| Position | **^13^C** | **^1^H** |
| 1 | 178.1, (C=O) |  |
| 2 | 33.7, (CH_2_) | 2.35, br.*t* (4.0, 8.0), 2H |
| 3 | 31.9, (CH_2_) | 1.63, *m*, 2H |
| 4-19 | 29.7 – 22.7, (CH_2_) | 1.25, br.*s*, 32H |
| 20 | 14.1, (CH_3_) | 0.88, *t*, (8.0), 3H |
| - |  | 10.00, br*.s*, OH |

Compound 3 (arjunolic acid) was obtained as a white powder (15 mg) and responded positively to the Liebermann - Büchard test characteristic of triterpenoids. The NMR spectra (^1^H, ^13^C, ASAP-HMQC, COSY and HMBC) are presented in Figure 11-15. The ^13^C-NMR spectrum (Table 3 Figure 12) had a total number of 30 carbons of which six were methyl carbon signals at *δ* 13.7 (C-23), 16.8 (C-25), 16.9 (C-26), 25.7 (C-27), 32.1 (C-29), 23.0 (C-30) and six downfield carbon signals at 178.6 (C-28), 144.0 (C-13), 121.5 (C-12), 75.5 (C-3), 67.4 (C-2) and 63.9 (C-24) characteristic for olean-12-ene triterpenoid [29] bearing one carboxylic acid and three hydroxyl groups. This assumption was substantiated by the presence of some characteristic proton signals on the ^1^H-NMR spectrum (Table 2, Fig 6) at *δ* 5.17 (br.*s*, COOH-28), 3.47 (*m*, H-2), 3.17 (*m*, H-3), 2.74 (br.*d*, 8.0 Hz, H-9), 0.54 (*s*, Me-23), 0.87 (*s*, Me-25), 0.71 (Me-26), 1.10 (*s*, Me-27), 0.87 (*s*, Me-29) and 0.91 (*s*, Me-30). All the data above along with the HSQC, HMBC and COSY were in agreement with those reported for arjunolic acid (Fig. 11-15) [10], a constituent of the core wood of *Terminalia arjuna* [11].

Table S3 ^1^H- (400 MHz) and ^13^C- (100 MHz) NMR data of arjunolic acid in DMSO-*d_6_*, (*δ* in ppm, *J* in Hz)

| Position | ^13^C | ^1^H |
| --- | --- | --- |
| 1 | 45.7 (CH_2_) | - |
| 2 | 67.4 (CH) | 3.47 *m* |
| 3 | 75.5 (CH) | 3.17 *m* |
| 4 | 42.5 (C) |  |
| 5 | 45.4 (CH) | - |
| 6 | 17.5 (CH_2_) | - |
| 7 | 32.9 (CH_2_) | - |
| 8 | 37.4 (C) |  |
| 9 | 40.8 (CH) | 2.74 *bd* (8.0) |
| 10 | 41.4 (C) |  |
| 11 | 23.4 (CH_2)_ | - |
| 12 | 121.5 (CH) | 5.17 *brs* |
| 13 | 144.0 (C) |  |
| 14 | 47.1 (C) |  |
| 15 | 30.4 (CH_2_) | - |
| 16 | 27.2 (CH_2_) | - |
| 17 | 46.7 (C) |  |
| 18 | 41.4 (CH) | - |
| 19 | 46.0 (CH) | - |
| 20 | 30.4 (CH) | - |
| 21 | 33.3 (CH_2_) | - |
| 22 | 31.9 (CH_2_) | - |
| 23 | 13.7 (CH_3_) | 0.54 *s* |
| 24 | 63.9 (CH_2_) | 3.29 *d* (8.0), 3.03 *d* (8.0) |
| 25 | 16.8 (CH_3_) | 0.87 *s* |
| 26 | 16.9 (CH_3_) | 0.71 *s* |
| 27 | 25.7 (CH_3_) | 1.10 *s* |
| 28 | 178.6 (C) |  |
| 28-COOH |  | 12.00 *brs* |
| 29 | 32.1 (CH_3_) | 0.87 *s* |
| 30 | 23.0 (CH_3_) | 0.91 *s* |

Figure S1**.** ^1^H-NMR spectrum of n-tetracosanol

Figure S2**.** ^13^C-NMR spectrum of n-tetracosanol

Figure S3**.** ASAP-HMQC-NMR spectrum of n-tetracosanol

Figure S4**.** COSY-NMR spectrum of n-tetracosanol

Figure S5. HMBC-NMR spectrum of n-tetracosanol

Figure S6. ^1^H-NMR spectrum of eicosanoic acid

Figure S7. ^13^C-NMR spectrum of eicosanoic acid

Figure S8. ASAP-HMQC-NMR spectrum of eicosanoic acid

Figure S9. COSY-NMR spectrum of eicosanoic acid

Figure S10. HMBC-NMR spectrum of eicosanoic acid

Figure S11. ^1^H-NMR spectrum of arjunolic acid

Figure S12. ^13^C-NMR spectrum of arjunolic acid

Figure S13. ASAP-HMQC-NMR spectrum of arjunolic acid

Figure S14. COSY-NMR spectrum of arjunolic acid

Figure S15. HMBC-NMR spectrum of arjunolic acid

**C1=n-Tetracosanol, C2=Eicosanoic acid and C3=Arjunolic acid**

Figure S16. Percentage cell viability of C3A cells exposed to different concentrations of compounds isolated from *C. microphyllum*

**A**

**C1=n-Tetracosanol, C2=Eicosanoic acid and C3=Arjunolic acid**

**B**

Figure S17. Percentage DPPH free radical scavenging activity of compounds isolated from *C. microphyllum* (A) and ascorbic acid (B)
